# Episiotomy and perineal trauma during childbirth in primiparous women: associations with anxiety, quality of life, vaginal and sexual symptoms in the first year postpartum

**DOI:** 10.3389/fmed.2025.1510417

**Published:** 2025-06-03

**Authors:** Maria Patricia Roman, Răzvan Ciortea, Stergios K. Doumouchtsis, Andrei Mihai Măluţan, Carmen Elena Bucuri, Oana Mihaela Oltean, Cristina Mihaela Ormindean, Viorela Elena Suciu, Ionel Daniel Nati, Denisa Rus, Dan Mihu

**Affiliations:** ^1^2nd Obstetrics and Gynecology Clinical Section, Cluj County Emergency Clinical Hospital, Cluj-Napoca, Romania; ^2^Department of Mother and Child, “Iuliu Haţieganu” University of Medicine and Pharmacy, Cluj-Napoca, Romania; ^3^Department of Obstetrics and Gynecology, Epsom and St. Helier University Hospitals NHS Trust, Epsom, United Kingdom; ^4^St. George’s University of London, Cranmer Terrace, London, United Kingdom; ^5^Laboratory of Experimental Surgery and Surgical Research “N.S. Christeas,” National and Kapodistrian University of Athens, Medical School, Athens, Greece; ^6^School of Medicine, American University of the Caribbean, Cupecoy, Sint Maarten; ^7^School of Medicine, Ross University, Miramar, FL, United States; ^8^Military Emergency Hospital “Dr. Constantin Papilian”, Cluj-Napoca, Romania

**Keywords:** childbirth perineal trauma, episiotomy, anxiety, ICIQ-VS, patient-reported outcomes, vaginal symptoms, sexual symptoms, quality of life

## Abstract

**Introduction:**

Childbirth-related pelvic floor trauma is prevalent among primiparous women and can lead to significant physical and psychological consequences. While the impact of pelvic floor trauma on physical outcomes has been studied, the relationship between anxiety caused by such trauma and long-term patient-reported outcomes (PROs) such as vaginal symptoms, sexual function, and quality of life (QoL) remains underexplored. This study aims to fill this gap by investigating the association between anxiety induced by pelvic floor trauma during childbirth and these key PROs.

**Methods:**

This prospective longitudinal cohort study analyzed data from 175 nulliparous women who delivered at term a singleton fetus in cephalic presentation and sustained some form of perineal trauma. Anxiety levels were assessed at two time points: during labor and at 12 months postpartum, using a single-item 10-point Likert scale. The other PROs were measured using the International Consultation on Incontinence Questionnaire-Vaginal Symptoms tool (ICIQ-VS).

**Results:**

Findings revealed that higher anxiety scores at birth were associated with elevated anxiety levels at 12 months postpartum and correlated significantly with increased vaginal symptoms, sexual symptoms, and QoL. Notably, while anxiety was linked to negative physical outcomes, higher anxiety scores were also associated with improved perceived QoL, suggesting the potential role of coping mechanisms in response to childbirth trauma as well as the need for future studies using more specialized anxiety tools.

**Conclusion:**

The study underscores the intricate relationship between psychological distress and physical health outcomes in postpartum women. Addressing both anxiety and physical symptoms through personalized care strategies may enhance recovery and overall wellbeing. Future research should explore effective interventions to mitigate anxiety, evaluate resilience and improve PROs in this population.

## 1 Introduction

Childbirth pelvic floor trauma refers to injuries affecting different structures of the pelvic floor (1), especially among primiparous women. Muscle, nerve and connective tissue damage may occur at the level of the perineal or vaginal tissues, potentially involving the anal sphincter complex and the rectum. Episiotomy and spontaneous perineal lacerations (grades I to IV) are all forms of pelvic floor trauma and may occur either in isolation or in combination, depending on the circumstances of childbirth.

The global incidence of pelvic floor trauma in primiparous patients exceeds 91% (1, 2), affecting women worldwide. Several factors, such as increased tissue resistance, longer labor duration, the need for assisted delivery contribute to the higher incidence of pelvic floor trauma in primiparous compared to multiparous women. The most commonly performed obstetric surgical procedure in the second stage of labor is episiotomy (3), being a leading cause of childbirth perineal trauma, while aiming to prevent trauma. The overall prevalence of this procedure significantly differs, ranging from 2 to 86% (4–6). Second-degree perineal tears occur in approximately 40% of primiparous women, while third- and fourth-degree tears happen in around 6% of births within this group (7).

The consequences of pelvic floor childbirth trauma can vary greatly, often having a profound impact on women, both physically and psychologically. Pelvic floor injuries can cause both immediate and long-term complications such as: hemorrhage, infection, wound dehiscence, abnormal scarring, pelvic organ prolapse, chronic pelvic pain, urinary or fecal incontinence, dyspareunia, etc. (8). While the physical consequences of pelvic floor trauma are well-documented, the psychological burden of these injuries is not to be neglected. The trauma sustained at childbirth often co-exists with anxiety, depression, sleep disorders and requires recognition as well as a personalized, multidisciplinary care plan (9).

However, the relationship between childbirth-related anxiety and long-term patient-reported outcomes (PROs) such as vaginal symptoms, sexual function, and overall QoL remains underexplored (10, 11). Understanding these relationships is critical for improving postpartum care and ensuring the wellbeing of women. Anxiety, whether experienced during childbirth or persisting into the postpartum period, can exacerbate physical symptoms, hinder recovery, and negatively impact QoL. Conversely, unresolved physical symptoms, such as vaginal laxity, vulvodynia or dyspareunia, can contribute to psychological distress, creating a cyclical relationship between physical and mental health.

To assess these outcomes, patient reported outcome measures (PROMs) are essential. PROMs are standardized questionnaires used in healthcare and research used to collect information directly from patients about their health and provide valuable insight on how patients perceive their own state. Several tools such as International Consultation on Incontinence Questionnaire (ICIQ)-vaginal symptoms (VS) (12), ICIQ-urinary incontinence (UI) (13)], Pelvic Floor Disability Index (PFDI) (14) have been developed and validated in the domain of pelvic floor disorders (10, 15–19). For the purpose of this study, the ICIQ-VS tool was used to quantify the symptoms of the included patients. This tool is a validated (12, 15–20) “self-completion questionnaire for comprehensive assessment of the severity and impact of vaginal symptoms, related sexual matters, particularly those attributed to pelvic organ prolapse” (12). Additionally, ICIQ-VS also quantifies the impact of the above-mentioned symptoms on QoL. The ICIQ-VS is included within the broader ICIQ spectrum of tools (21), which are widely recognized for their reliability and validity in measuring PROs related to pelvic floor dysfunction.

The current study aims to address the knowledge gap regarding the interconnections between anxiety induced by pelvic floor trauma during childbirth and the above-mentioned PROs, as well as between different PROs, at 12 months postpartum, with a focus on vaginal symptoms, sexual symptoms, and QoL. The long-term follow-up allowed for a clearer understanding of how psychological factors interact with physical outcomes in the postpartum period, offering valuable insights for personalized, women centered and holistic care strategies.

## 2 Materials and methods

### 2.1 Study design

This prospective longitudinal cohort study analyzed data collected from women who delivered from January 2022 to September 2023 in a secondary maternity hospital. Women were eligible to participate if they were aged 18 years or older, nulliparous, had given birth vaginally to a term fetus in cephalic presentation, and had sustained some form of perineal trauma during childbirth. Restricting this study to women who delivered their first fetus in cephalic presentation, helped control for the confounding effects of parity and fetal presentation.

Consent for study participation was sought upon admission to the labor ward. Participants were informed that their involvement in the study had two stages: the first one during labor, and the second at 12 months postpartum. Patients were made aware that they can freely withdraw at any time. Continued consent was assumed by their responses to the questionnaires delivered during the 12-month postpartum follow-up.

Once the woman has signed the informed consent, the following baseline data were collected upon enrollment: age, residential setting and body mass index (BMI). Women who underwent a cesarean section were excluded from the analysis. After delivery, data on the type of perineal laceration sustained, the use of epidural analgesia in labor, and use of instrumental delivery were also recorded.

### 2.2 Outcomes

The primary outcome measured in this study was patient-reported anxiety induced by perineal trauma at two different time points: during labor and at 12 months postpartum. Anxiety scores were measured using a single item 10-point Likert scale, where participants were asked to rate their anxiety from 1 to 10, with 1 indicating “no anxiety” and 10 indicating “the highest level of anxiety.” This scale was chosen for its simplicity, ease of use, and widespread application in clinical and research settings for measuring subjective experiences of patients. The 10-point Likert scale has been validated in previous research and has shown adequate performance for assessing anxiety levels (22). During labor, participants were asked, “How anxious do you feel about the possibility of sustaining some type of perineal trauma at birth, including episiotomy?”; At 12 months postpartum, they were asked, “How anxious do you feel now about the perineal trauma sustained at birth?” Patients were provided with the following definition of anxiety related to childbirth perineal trauma: “feelings of worry, fear, or nervousness about the possibility of tearing or needing an episiotomy during childbirth. This anxiety may also stem from concerns about pain, recovery, or long-term effects on physical comfort, body image, or sexual function following childbirth.”

Secondary outcomes of interest were PROs as obtained from the ICIQ-VS, which was administered to patients during a 12-month follow-up phone call conducted by a member of the research team. This tool is a 14-item questionnaire designed to assess three domains: vaginal symptoms (referring to vaginal laxity, reduced sensation, protruding masses, dryness, impaired fecal evacuation, and pain), sexual matters (impact on relationship and sexual function) and their effect on QoL. Each domain includes specific questions scored on a rating system, allowing for quantitative assessment of symptom severity. The ICIQ-VS questionnaire has been validated across diverse populations and has shown varying internal consistency, with Cronbach’s alpha from 0.64 (19) to 0.79 (18) for vaginal symptoms and from 0.69 (19) to 0.86 (15) for sexual matters.

### 2.3 Risk of bias considerations

The research team members conducting the 12-month follow-up assessments on anxiety, vaginal and sexual symptoms, and QoL, were blinded to the participants’ delivery details (e.g., type of perineal trauma) to reduce observer bias. This ensured that data collection remained objective and unbiased by prior knowledge of patient outcomes.

To minimize measurement bias, standardized and validated tools (single item 10-point Likert scale and ICIQ-VS) were used to assess outcomes. The consistency in measurement scales helped ensure reliable data collection across participants.

### 2.4 Safety considerations

All the interventions that the participants underwent were part of their routine care at birth. No special treatments were administered to enrolled individuals. However, potential benefits and disadvantages might have arisen from participation to the study.

A potential benefit was that the member of the research team investigating the anxiety could identify those patients with high anxiety scores at birth. Any women flagged as particularly anxious were offered support by the research team, who guided them toward appropriate counseling.

Disadvantages included potential shame and discomfort involved when addressing sensitive topics such as vaginal symptoms or sexual matters during follow-up phone calls. In a research setting, addressing these issues requires strategies that are thoughtful and empathetic. To overcome these issues, the following approaches were used: pre-call information was provided in advance (upon enrollment), explaining the nature and the purpose of the questions; all participants were assured that all responses are confidential, and data would be anonymized. Participants were offered the option to withdraw from the study if they felt uncomfortable or to skip questions they did not wish to answer.

### 2.5 Statistical analysis

The sample of patients included in the dataset was described using means and standard deviations for continuous variables such as maternal age, neonatal birthweight, maternal BMI. Counts and proportions were used for categorical variables such as use of epidural analgesia, residential setting (rural vs. urban), type of delivery (spontaneous vs. instrumental), and type of perineal trauma (episiotomy, midline episiotomy, spontaneous laceration or their combinations).

The Mann-Whitney test was used to compare the scores of primary and secondary patient-reported outcomes, namely anxiety, vaginal symptoms, sexual symptoms, and QoL across different groups, ensuring valid comparisons. To explore potential correlations between the studied variables, the non-parametric Spearman correlation coefficient (r) and the corresponding two-tailed *p*-values (with a 95% confidence interval) were calculated.

Missingness in variables as addressed using the pairwise deletion approach. Although this approach led to varying sample sizes across different analyses, it had the advantage of excluding only cases where specific data points were missing, thus maximizing the use of available data.

Power calculations were performed prior to data collection to ensure the study was sufficiently powered to detect significant differences between groups using G*Power 3.1.9.7 tool (23, 24). Based on a desired power of 80% (1-β = 0.80) and a significance level of α = 0.05, a sample size of 128 participants was estimated to detect a medium effect size (Cohen’s d = 0.5) for comparisons using the Mann-Whitney *U*-test, and a sample size of 29 participants was estimated to detect a medium effect size (Cohen’s *d* = 0.5) for using Spearman’s correlation. Power calculations accounted for the possibility of missing data, and adjustments were made accordingly during analysis.

Statistical analyses were conducted using GraphPad Prism 9.1.0.

Internal consistency of the ICIQ-VS tool was evaluated by calculating Cronbach’s alpha coefficient.

## 3 Results

One hundred seventy-five primiparous women who delivered between January 2022 and September 2023 were included in this analysis. The characteristics of the included patients are summarized in Table 1.

**TABLE 1:** Characteristics of women included in the dataset.

Characteristic	*N* = 175
Maternal age in years, mean (SD)	26.6 (4.67)
Birthweight in grams, mean (SD)	3239.5 (411.16)
Maternal BMI (kg/m^2^), mean (SD)	28.47 (4.29)
Epidural analgesia use, n (%)	84 (48)
Rural residential setting, n (%)	80 (45.71)
Urban residential setting, n (%)	99 (56.57)
Spontaneous delivery, n (%)	167 (95.4)
Instrumental delivery, n (%)	8 (4.57)

All patients included in our analysis sustained some sort of perineal trauma. Most women (81.14%) underwent a medio-lateral episiotomy during birth, while only 7.34% suffered a midline episiotomy. Combined, episiotomy and spontaneous lacerations, occurred in only 4.58% of cases, as shown in Table 2. None of the women experienced grade 3 or 4 perineal lacerations. Given the high prevalence of episiotomies (94%) in our cohort, our assessment focused on episiotomy-related outcomes rather than other types of childbirth-related perineal trauma.

**TABLE 2:** Types and prevalence of perineal trauma.

Type of perineal trauma	N (%)
Medio-lateral episiotomy	142 (81.14)
Midline episiotomy	13 (7.43)
Episiotomy + grade 1 laceration	4 (2.29)
Episiotomy + grade 2 laceration	4 (2.29)
Episiotomy + grade 3 or 4 laceration	0
Midline episiotomy + grade 1-4 laceration	0

As shown in Table 3, no statistically significant difference between anxiety scores at birth were observed across the five compared groups, with *p*-values ranging from 0.15 to 0.85. When analyzing the anxiety score reported by women across the compared groups at 12 months postpartum, the only statistically significant difference emerged between women who underwent episiotomy and those who did not (*p* = 0.04), with the former reporting higher anxiety scores. For the remaining comparisons shown in Table 3, *p*-values ranged from 0.34 and 0.70.

**TABLE 3:** Comparison of anxiety score at birth and at 12 months postpartum across different groups.

	Anxiety score at birth			Anxiety score at 12 months postpartum		
**Group**	**n**	**Mean ± SD**	***p*-value** **(Mann-Whitney statistic test)**	**n**	**Mean ± SD**	***p*-value** **(Mann-Whitney statistic test)**
Epidural analgesia	82	4.11 ± 3.44	0.85	68	3.02 ± 3.33	0.53
No epidural analgesia	93	3.96 ± 3.17		71	3.25 ± 3.24	
Episiotomy				114	3.45 ± 3.40	**0.04**
No episiotomy				25	1.72 ± 2.151	
Women < 25 years old	54	3.88 ± 3.19	0.70	38	3.63 ± 3.67	0.51
Women ≥ 25 years old	121	4.09 ± 3.34		101	2.96 ± 3.11	
Rural residential setting	78	4.17 ± 3.27	0.15	59	3.47 ± 3.42	0.34
Urban residential setting	97	3.91 ± 3.32		80	2.9 ± 3.16	
BMI < 30	123	3.80 ± 3.21	0.19	99	3.15 ± 3.22	0.70
BMI ≥ 30	52	4.57 ± 3.45		40	3.12 ± 3.44	

Table 4 compares the scores for vaginal symptoms, sexual symptoms, and QoL. The results suggest no significant differences in vaginal symptoms, sexual symptoms and QoL scores between women who received epidural analgesia and those who did not, with *p*-values of 0.39, 0.30, and 0.95, respectively. Similarly, no differences in vaginal symptoms, sexual symptoms, and QoL scores were noted based on episiotomy, age, residential setting or BMI, with all the corresponding *p*-values being greater than 0.05.

**TABLE 4:** Comparison of vaginal symptoms, sexual symptoms and QoL scores across different groups.

	Vaginal symptoms score			Sexual symptoms score			Quality of life score		
**Group**	***n***	**Mean ± SD**	***p*-value** **(Mann-Whitney statistic test)**	***n***	**Mean ± SD**	***p*-value** **(Mann-Whitney statistic test)**	***n***	**Mean ± SD**	***p*-value** **(Mann-Whitney statistic test)**
Epidural analgesia	74	16.12 ± 17.78	0.39	71	33.77 ± 44.73	0.30	74	2.20 ± 2.39	0.95
No epidural analgesia	83	13.61 ± 15.97		82	28.45 ± 39.87		83	2.31 ± 2.54	
Episiotomy	128	15.94 ± 17.83	0.16	124	29.78 ± 42.13	0.26	128	2.39 ± 2.60	0.49
No episiotomy	29	9.759 ± 10.33		29	35.79 ± 42.59		29	1.69 ± 1.67	
Women < 25 years old	58	14.02 ± 18.93	0.24	40	20.20 ± 35.81	0.081	43	2.46 ± 2.45	0.30
Women ≥ 25 years old	99	15.25 ± 15.57		113	34.72 ± 43.68		114	2.18 ± 2.48	
Rural residential setting	67	18.04 ± 20.05	0.15	63	35.30 ± 44.37	0.25	67	2.43 ± 2.57	0.41
Urban residential setting	90	12.38 ± 13.60		90	27.86 ± 40.47		90	2.13 ± 2.39	
BMI < 30	111	14.85 ± 16.27	0.69	110	29.85 ± 40.26	0.89	88	2.52 ± 2.51	0.50
BMI ≥ 30	46	14.67 ± 18.34		43	33.67 ± 47.00		46	2.32 ± 2.66	

The potential relationship between anxiety at birth and at 12 months postpartum was further explored in the full cohort of participants to assess the extent to which initial anxiety predicted future anxiety, as well as related vaginal or sexual symptoms and changes in QoL following perineal trauma sustained during birth. Table 5 indicates that anxiety scores at birth were significantly correlated with anxiety scores at 12 months postpartum (*p* < 0.0001). In addition, significant correlations were found between anxiety and vaginal symptoms (*p* = 0.02 at birth, *p* = 0.001 at 12 months postpartum), sexual symptoms (*p* = 0.013 at birth, *p* = 0.003 at 12 months postpartum) and QoL (*p* = 0.0002 at birth, *p* < 0.0001 at 12 months postpartum).

**TABLE 5:** *P*-values indicating significant correlations between the studied variables.

Variable	Anxiety at birth	Anxiety at 12 months postpartum
Anxiety at birth		<0.0001
Anxiety at 12 months postpartum	<0.0001	
VS	0.02	0.001
SS	0.01	0.003
QoL	0.0002	<0.0001

VS, vaginal symptoms; SS, sexual symptoms.

**FIGURE 1:**
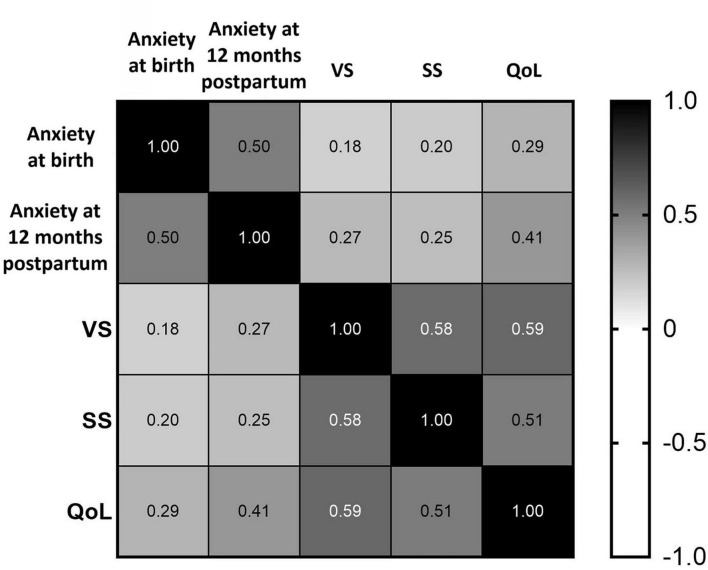
Heatmap of correlation matrix between anxiety, vaginal symptoms, sexual symptoms, and QoL scores.

To illustrate the interrelationship between anxiety and key postpartum outcomes, a heatmap was generated to visualize the correlation matrix between anxiety, vaginal symptoms, sexual symptoms and QoL. The heatmap depicted in Figure 1 highlights negligible correlations between anxiety scores at birth and vaginal symptoms (*r* = 0.18). Weak correlations between anxiety and sexual symptoms (*r* = 0.20 at birth and *r* = 0.25 at 12 months postpartum), as well as between anxiety at 12 months postpartum and vaginal symptoms (*r* = 0.27) were demonstrated. The remaining correlations illustrated in Figure 1, between vaginal symptoms, sexual symptoms, and QoL, with *r*-values ranging from 0.51 to 0.59 were classified as strong. The strongest correlation was observed between vaginal symptoms and QoL (*r* = 0.59).

Cronbach’s alpha coefficients of the vaginal symptoms score and sexual matters score, indicating the internal consistency of the ICIQ-VS tool, were 0.65 and 0.87, respectively.

## 4 Discussion

### 4.1 Key findings

The results indicated significantly higher anxiety scores at 12 months postpartum in women who underwent episiotomy compared to those who did not. This observation may suggest that experiencing episiotomy may be associated with greater physical trauma or recovery challenges. Potential complications or dissatisfaction with birth experience may have also contributed to this finding. However, it is important to acknowledge that the non-episiotomy group represented only 4.58% of the cohort, potentially limiting the generalizability of this finding. The small sample size in this group increases the risk of sampling bias. Therefore, these results should be interpreted with caution. Despite the small sample size in our non-episiotomy group, the finding that women who underwent episiotomy reported higher anxiety levels at 12 months postpartum is consistent with previous literature suggesting that surgical interventions during childbirth may have a psychological impact. For example, a study by Opondo et al. (10) indicated that perineal injuries, including episiotomies, are associated with increased postpartum anxiety and even depressive symptoms, highlighting long-term psychological consequences (10). However, future studies with larger and more balanced cohorts are needed to determine whether the observed difference is robust across larger and more diverse populations.

Although episiotomy did make a difference in terms of anxiety scores, the present study also found that most differences between anxiety at birth and at 12 months postpartum were not significant when compared across the study groups: epidural vs. no epidural, age < 25 years vs. ≥ 25 years, rural vs. urban residential setting and BMI < 30 vs. ≥ 30. The lack of significant differences between these groups suggests that these factors may not have a strong influence on anxiety levels. A potential interpretation could include uniformity of experience, which means that women with different demographics may share similar experiences or coping mechanisms for pelvic floor childbirth trauma-related anxiety, regardless of their background. However, it is important to note that factors such as unmeasured confounders, individual differences in support systems, and personal resilience may influence the observed results.

When comparing vaginal symptoms, sexual symptoms and QoL scores among the same study groups, no significant differences emerged in the present study. These findings suggest that the demographic factors assessed may not play a critical role in influencing the outcomes studied. Factors such as psychological resilience and social support may have a more pronounced effect on the studied outcomes than the demographic variables analyzed. Research has demonstrated that psychosocial aspects can significantly influence postpartum recovery, including physical symptoms and overall wellbeing (25, 26).

When exploring the relationship between anxiety at birth and at 12 months postpartum within the full cohort of patients, we found that anxiety at birth was correlated with anxiety at 12 months postpartum. As anxiety levels at birth increase, anxiety levels at 12 months postpartum tend to increase as well. The persistence of anxiety over time may reflect unresolved psychological stressors related to the childbirth experience. Previous studies have suggested that high levels of anxiety during childbirth can lead to increased risks of postpartum anxiety (27, 28), with wider implications such as difficulties in couples’ emotional and sexual relationships (29), self-blaming or aggression toward others (30), impact on subsequent reproductive decisions (31–33) and, not ultimately, more health services use (27).

Moreover, both anxiety at birth and at 12 months postpartum were significantly and positively correlated with the other studied PROs, vaginal symptoms, sexual symptoms, and QoL in the present study. The PROs studied also correlated positively with each other. While it is understandable that higher anxiety scores positively correlate with increased vaginal and sexual symptoms, it is intriguing to note that patients who reported higher scores for anxiety, vaginal or sexual symptoms also reported higher QoL scores. These results partly align with previously published studies (10, 34), contributing to the growing body of knowledge that confirms that anxiety can exacerbate the perception of physical discomfort (34). The results that oppose previously published findings are the positive correlations between anxiety, vaginal and sexual symptoms and QoL. This could suggest that higher anxiety levels might not solely lead to negative outcomes; rather, individuals experiencing higher anxiety, vaginal and sexual symptoms may also engage in coping mechanisms or support systems that enhance their perceived QoL, warranting further investigation into the nuances of these relationships. Future studies may benefit from qualitative approaches to identify effective coping mechanisms used to mitigate the negative impact of anxiety as well as physical symptoms related to childbirth perineal trauma on QoL.

### 4.2 Limitations and strengths of the study

While the study presents important findings regarding the relationship between childbirth perineal trauma, anxiety and postpartum outcomes, it is essential to consider the identified limitations in future research.

First, it is important to recognize potential confounding factors that may have led to bias. Despite adjusting for key variables such as maternal age, BMI, residential setting, episiotomy, and the use of epidural analgesia, residual confounders may still exist. Factors such as socioeconomic status, pre-existing mental health conditions—baseline anxiety, and variations in social support systems were not accounted for, and they may have influenced the observed relationships between childbirth perineal trauma, anxiety and postpartum outcomes. Future research should consider these factors to further reduce the potential for residual confounding.

Second, the reliance on self-reported data could introduce response bias (35, 36). Although standardized measures like the ICIQ-VS were used, study participants may underreport or overreport symptoms based on personal perceptions or societal norms (37–39).

Third, not all groups had the same weight in the present study; some of them such as women younger than 25 years or with a BMI greater than 30 have been underpowered. The subgroups of women that had smaller sample sizes may have limited our ability to detect statistically significant differences in anxiety scores and other studied outcomes. As a result, the non-significant findings observed in those groups with smaller sample sizes should be interpreted with caution, as they may reflect insufficient power rather than the absence of an effect (40). Future studies with larger sample sizes are needed to validate these findings and better assess the potential differences between these subgroups.

Fourth, it is important to acknowledge that longitudinal studies face challenges related to missing data. In our case, this occurred due to the following reasons: participant dropout over time or incomplete responses during follow-up assessments, primarily stemming from feelings of shame related to the sensitive nature of the questionnaire topics. However, pairwise deletion method was used to mitigate data missingness which has the advantage of allowing the maximum use of available data by including all available cases for each analysis (41).

Lastly, the generalizability of findings should be used with caution when looking at specific types of childbirth perineal trauma. While the study provided valuable insights into childbirth perineal trauma-related anxiety and its association with PROs, it primarily focused on perineal trauma as a general category, with a significant proportion of participants experiencing episiotomy. Other important groups, such as those with obstetric anal sphincter injuries, midline episiotomy, or spontaneous lacerations were underrepresented. This limitation highlights the importance of detailed subgroup analyses in research related to childbirth trauma, as understanding the nuances of different injuries can significantly inform patient care and enhance individualized counseling.

While the limitations of this study highlight important areas for improvement, they also serve to underscore its strengths.

To the best of our knowledge, this study represents the first prospective longitudinal cohort investigation into specific PROs within a homogenous group of primiparous women. These women delivered at term, a singleton fetus in cephalic presentation and sustained childbirth pelvic floor perineal trauma. By focusing on a homogenous cohort, this study provides a unique perspective on the relationship between anxiety and the PROs measured one year after birth.

Additionally, the use of validated measures, such as the ICIQ-VS, allowed for meaningful, quantitative, comparisons across outcomes. The consistency of the ICIQ-VS allows for comparisons not only within the study cohort but also with findings from other research, facilitating a more comprehensive understanding of how pelvic floor trauma and anxiety impact women’s health postpartum.

Furthermore, the prospective design of the study enables a more nuanced understanding of how anxiety evolves over time, contributing valuable insights into postpartum mental health. It is also interesting to point out that the study opens new perspectives into future research by looking at anxiety not only as a negative aspect but also as a motivator to seek strategies that lead to improved outcomes.

Overall, the strengths of this study complement their limitations and pave the way for future research that can build upon these findings to improve maternal health outcomes.

### 4.3 Recommendations for clinical practice

In light of these findings and limitations, it is essential to reflect on how they can inform clinical practice, decision-making processes and individualized patient care. This section offers practical suggestions aimed at improving outcomes for women experiencing childbirth perineal trauma, with a particular focus on anxiety management and tailored postpartum support.

Although drawing conclusions about spontaneous tears versus episiotomy is challenging due to the sample distribution in our groups, our findings underscore the importance of minimizing unnecessary interventions to reduce the associated anxiety (42). A conservative approach to episiotomy may help reduce both physical and psychological burden during the postpartum period. Encouraging women to be part of the decision-making process regarding intervention during childbirth may help to reduce feelings of helplessness and anxiety. Moreover, engaging in pre-labor preparation techniques, such as perineal massage (7) and antenatal education courses (43), could also have a benefic impact. Perineal massage has been shown to reduce the risk of perineal trauma (7), while antenatal courses may help women feel more empowered and less anxious about childbirth (43). Women who undergo episiotomy or experience perineal trauma should receive individualized postpartum care, including psychological support and pelvic floor rehabilitation, to address both physical symptoms and anxiety.

Additionally, being vigilant in addressing postpartum anxiety, particularly in women who undergo episiotomy is important. Early identification and intervention, such as counseling or behavioral therapy, may help mitigate long-term psychological distress and improve overall QoL during the postpartum period (44).

Finally, establishing seamless communication among multidisciplinary team members, including obstetricians, midwives, pelvic floor therapists, and mental health professionals is essential to provide holistic care for women experiencing childbirth-related trauma.

## Conclusion

This study provides insights into the relationship between pelvic floor childbirth trauma-related anxiety and PROs in primiparous women. The findings demonstrate that anxiety at birth is associated with higher anxiety levels at 12 months postpartum. Anxiety at two different time points, at birth and at 12 months postpartum, also correlated with increased vaginal and sexual symptoms and overall QoL. Additionally, vaginal symptoms, sexual symptoms and QoL were significantly correlated with one another. The fact that women who underwent episiotomy reported higher anxiety levels at 12 months postpartum compared to those who did not is an important contribution to the literature, but it must be interpreted cautiously due to the small proportion of non-episiotomy cases. These results underscore the intricate relationship between psychological and physical health during the postpartum period, suggesting that anxiety, as well as physical and sexual symptoms, may act as catalysts for women to seek coping strategies for the effects of childbirth trauma, ultimately leading to QoL enhancement.

## Data Availability

The original contributions presented in this study are included in this article/supplementary material, further inquiries can be directed to the corresponding author.
